# Reducing the standard serving size of alcoholic beverages prompts reductions in alcohol consumption

**DOI:** 10.1111/add.14228

**Published:** 2018-05-14

**Authors:** Inge Kersbergen, Melissa Oldham, Andrew Jones, Matt Field, Colin Angus, Eric Robinson

**Affiliations:** ^1^ Department of Psychological Sciences University of Liverpool Liverpool UK; ^2^ UK Centre for Tobacco and Alcohol Studies UK; ^3^ School of Health and Related Research The University of Sheffield Sheffield UK

**Keywords:** Alcohol consumption, alcohol policy, drinking environment, nudge, portion size, serving size

## Abstract

**Aims:**

To test whether reducing the standard serving size of alcoholic beverages would reduce voluntary alcohol consumption in a laboratory (study 1) and a real‐world drinking environment (study 2). Additionally, we modelled the potential public health benefit of reducing the standard serving size of on‐trade alcoholic beverages in the United Kingdom.

**Design:**

Studies 1 and 2 were cluster‐randomized experiments. In the additional study, we used the Sheffield Alcohol Policy Model to estimate the number of deaths and hospital admissions that would be averted per year in the United Kingdom if a policy that reduces alcohol serving sizes in the on‐trade was introduced.

**Setting:**

A semi‐naturalistic laboratory (study 1), a bar in Liverpool, UK (study 2).

**Participants:**

Students and university staff members (study 1: *n* = 114, mean age = 24.8 years, 74.6% female), residents from local community (study 2: *n* = 164, mean age = 34.9 years, 57.3% female).

**Interventions and comparators:**

In study 1, participants were assigned randomly to receive standard or reduced serving sizes (by 25%) of alcohol during a laboratory drinking session. In study 2, customers at a bar were served alcohol in either standard or reduced serving sizes (by 28.6–33.3%).

**Measurements:**

Outcome measures were units of alcohol consumed within 1 hour (study 1) and up to 3 hours (study 2). Serving size condition was the primary predictor.

**Findings:**

In study 1, a 25% reduction in alcohol serving size led to a 20.7–22.3% reduction in alcohol consumption. In study 2, a 28.6–33.3% reduction in alcohol serving size led to a 32.4–39.6% reduction in alcohol consumption. Modelling results indicated that decreasing the serving size of on‐trade alcoholic beverages by 25% could reduce the number of alcohol‐related hospital admissions and deaths per year in the United Kingdom by 4.4–10.5% and 5.6–13.2%, respectively.

**Conclusions:**

Reducing the serving size of alcoholic beverages in the United Kingdom appears to lead to a reduction in alcohol consumption within a single drinking occasion.

## Introduction

Alcohol consumption contributes to premature death and ill health 1, and alcohol‐related harm places a substantial burden on society 2. Approximately 25% of alcohol consumers in England drink at higher‐risk levels, and 20% of high‐risk drinkers attempt to reduce their alcohol consumption 3. However, attempts to cut down do not lead often to actual reductions in alcohol consumption 4, 5. Therefore, changes to the environment that make it easier for people to drink less could have a substantial impact on public health 6, 7.

One potential environmental influence on alcohol consumption that is yet to be examined is serving size. Nutrition research shows consistently that portion sizes affect how much a person eats 8, 9. People eat more if they are given a relatively large portion of food compared to smaller portions, but they do not fully compensate for this by eating less afterwards 10. Similarly, people drink more if they are served a large non‐alcoholic beverage with their meal compared to a smaller serving of that beverage 11, 12.

There is a small amount of evidence indicating that the way that alcohol is served may affect drinking behaviour. A field study showed that the size of glass that people drink from may affect wine consumption 13, although this finding was not replicated fully at another venue 14. However, the effect that alcoholic beverage serving size has on alcohol consumption has not been examined. Given that serving size has a robust effect on food intake, and consumers do not appear to compensate later for changes in food serving size, we hypothesized that the serving size of alcohol beverages may have a causal effect on voluntary alcohol consumption. If alcoholic beverage serving size does indeed have a causal influence on alcohol consumption, then reductions to standard serving sizes could be an effective way of decreasing population‐level alcohol consumption and harm.

We aimed to investigate if reducing the serving size of alcoholic beverages would reduce alcohol consumption. In study 1, participants consumed alcohol from standard versus reduced serving sizes in a laboratory setting. The aims were to (1) compare total alcohol consumption from reduced servings and standard servings and (2) test whether there were any differences in the perceived ‘normality’ of the provided serving size between conditions. In study 2, participants consumed alcohol from standard versus reduced serving sizes in a local bar and subsequently reported any alcohol consumption that occurred after the study finished in order to examine whether participants compensated for the reduced serving sizes at later drinking occasions. The aims were to (1) compare total alcohol consumption from reduced servings with standard servings and (2) test whether there were any group differences in self‐reported alcohol consumption after the intervention period. We then used the findings from studies 1 and 2 to inform modelling of the effect of reductions in the serving size of on‐trade alcoholic beverages on alcohol‐related harm using the Sheffield Alcohol Policy Model (SAPM) 15. The aim was to estimate reductions in alcohol‐attributable deaths and hospital admissions as a result of serving size reductions.

## Study 1

## Methods

### Design

Pairs of participants attended a laboratory session, and both members of the pairs were randomized to receive alcoholic beverages in standard versus reduced serving sizes. *Ad libitum* alcohol consumption was measured during the course of 1 hour. We used cluster‐randomization to ensure that participants were blind to the experimental manipulation. We aimed to recruit a minimum of 50 participants per condition to have sufficient power to detect a medium to large effect size (*d* = 0.57) in a two‐tailed *t*‐test (α = 0.05) at 80% power, based on the effect sizes reported by Zlatevska *et al*. 8. We did not account formally for potential clustering in our sample size calculation, but we recruited slightly over the required minimum to increase power.

### Participants

One hundred and fourteen participants were recruited in pairs (57 pairs) from students and staff of the University of Liverpool. Participant pairs knew each other and were eligible if they were aged 18 years or older, consumed alcohol regularly (at least 10 UK units per week; 1 UK unit = 8 g of ethanol) and had a breath alcohol content (BAC) of zero upon arrival in the laboratory. Pairs were not constrained with regard to their gender composition. However, after testing 102 participants, there appeared to be a gender imbalance throughout conditions. We then stratified randomization by the pairs’ gender composition for the remaining participants to attenuate the gender imbalance. The study received ethical approval from the University of Liverpool ethics committee. Testing took place between July 2015 and March 2016.

### Serving size

**Figure 1:**
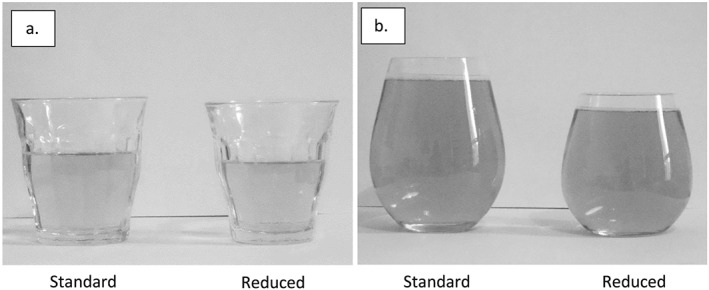
Study 1. Glassware used to serve wine (a) and beer/cider (b) in the standard and reduced serving conditions

Pairs of participants were assigned randomly to a standard serving size condition or a reduced serving size condition (between‐subjects). Participants had access to three types of alcoholic beverage: Magners cider [4.5% alcohol by volume (ABV)], Sol lager (4.5% ABV) and Isla Negra Sauvignon Blanc white wine (12.5% ABV). The beverages in the standard serving size condition contained 2.07 units per serving, which is equivalent to a typical UK serving of beer or wine. Beverages in the reduced serving size condition contained 1.55 units (a 25% reduction). See Table 1 for volume served and glassware capacity for the different drink types. The glasses used in the two conditions were of the same shape and width (Fig. 1), which resulted in glasses looking comparably full in both conditions.

**Table 1:** Studies 1 and 2: volume served and glassware capacity in the standard and reduced serving size condition.

Study	Drink type	Serving size condition	Volume served (ml)	Glass capacity (ml)
Study 1	Wine	Standard	165	310
		Reduced	125	250
	Beer/cider	Standard	460	530
		Reduced	345	370
Study 2	Wine	Standard	175	245
		Reduced	125	195
	Beer/cider	Standard	568	568
		Reduced	379	379

### Procedure and measures

The experiment took place on weekdays between 12:00 and 17:30 hours in a semi‐naturalistic laboratory designed to mimic a home environment that included a sofa, soft furnishings and a television. Participants were told that they were taking part in a study examining how social drinking affects opinions. After providing informed consent, participants’ blood alcohol content (BAC) and body weight were measured. Pairs of participants were allocated randomly to the serving size condition. The researcher was not blind to allocation. Participants were asked to order one alcoholic beverage at the start of the study and consume at least some of it. After this, they could order more beverages at any time during the study by pressing a serving button to notify the experimenter. To prevent adverse events, the experimenter monitored participants’ alcohol consumption via webcam to ensure that participants consumed no more than 0.8 g of alcohol per kg of body weight. After participants ordered their first beverage, to corroborate the cover story they completed an attitudes questionnaire on religion and human rights. The experimenter then began showing a 1‐hour TV programme on religion and human rights 16. Thirty minutes into the programme, the experimenter returned with another attitudes questionnaire and asked whether participants would like another beverage. After 1 hour, the experimenter returned with a final attitudes questionnaire. Participants then completed a questionnaire battery with (1) an open‐ended question to assess what participants thought the aims of the study were; (2) two 5‐point Likert items to investigate whether participants considered their own alcohol consumption during the study and the provided serving size to be ‘normal’ (anchors ‘strongly disagree’ and ‘strongly agree’); (3) the Restrict subscale of the Temptation and Restraint Inventory (TRI 17) as a measure of motivation to reduce drinking; (4) the Alcohol Use Disorders Identification Test (AUDIT 18) as a measure of hazardous drinking; and (5) a single item to assess weekly alcohol consumption in UK units. Finally, participants were thanked and debriefed. Each session lasted approximately 1.5 hours and participants received shopping vouchers for £10 or course credit as reimbursement for their time. All participants completed the study.

### Data analysis

We calculated how much beer, cider and wine participants consumed by subtracting the volume of any leftover beverage from the amount of beverages that were ordered. The total alcohol consumption in UK units was calculated by multiplying the volume consumed of each beverage type (in litres) with the beverages’ ABV. Alcohol consumption was distributed normally. We used multi‐level regression modelling to evaluate the amount of alcohol consumed across conditions, while controlling for data clustering within participant pairs. To investigate whether the effect of serving size on alcohol consumption was robust, we also controlled for gender (between‐subjects factor), AUDIT scores and TRI Restrict scores (covariates), because these covariates are likely to influence alcohol consumption. Finally, we used multi‐level regression modelling to evaluate perceived normality of the serving size and the amount of alcohol that participants consumed across conditions. All analyses were conducted in SPSS version 24 19. Analyses not accounting for clustering are reported in the Supporting information, Tables S1 and S2.

## Results

### Alcohol consumption

**Figure 2:**
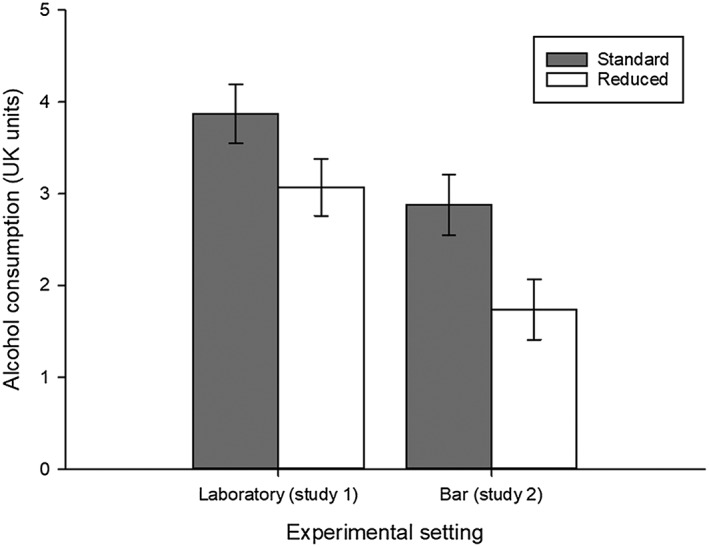
Studies 1 and 2. Mean alcohol consumption (UK units) in the standard and reduced serving size condition in a laboratory setting (study 1) and a real‐world setting (local bar, study 2). Bars represent raw means. Error bars indicate standard error of the mean (SEM)

Table 2 shows participant characteristics in both serving size conditions. The results of the multi‐level modelling showed a non‐significant reduction in alcohol consumption attributed to the reduced serving size condition [B = −0.80 (−1.69, 0.09), standard error (SE) = 0.44, *t*_(57)_ = 1.80, *P* = 0.08]. However, this reduction became significant when controlling for gender, AUDIT scores and TRI Restrict scores [B = −1.33 (−2.46, −0.20), SE = 0.57, *t*_(109.70)_ = 2.33, *P* = 0.02] (Table 3). Estimated means show that participants in the reduced serving size condition drank 20.7–22.3% less alcohol than participants in the standard serving size condition (Supporting information, Table S3, Fig. 2).

**Table 2:** Study 1: participant characteristics by serving size condition.

		Serving size condition	
	Total (n = 114)	Reduced (n = 60)	Standard (n = 54)
Age; mean (SD)	24.82 (10.48)	23.28 (8.61)	26.52 (12.08)
Gender; *n* male/female	29/85	11/49	18/36
AUDIT; mean (SD)	13.96 (6.06)	14.30 (6.52)	13.57 (5.54)
TRI Restrict; mean (SD)	9.70 (5.40)	9.55 (5.26)	9.87 (5.58)
UK units per week; mean (SD)	17.72 (12.27)	19.27 (12.99)	16.01 (11.29)

AUDIT = Alcohol Use Disorders Identification Test. AUDIT scores range between 0 and 40. TRI = Temptation and Restraint Inventory. TRI Restrict scores range between 3 and 21. SD = standard deviation.

**Table 3:** Study 1: unadjusted and adjusted multi‐level regression model with serving size predicting observed alcohol consumption (UK units); participants are clustered in pairs (level 2).

	Unadjusted (n = 114)			Adjusted (n = 114)		
	B (SE)	(95% CI)	P	B (SE)	(95% CI)	P
Fixed components						
Intercept	3.87 (0.32)	(3.23, 4.52)	< 0.001	3.99 (0.48)	(3.04, 4.95)	< 0.001
Serving size condition (reference: standard)	−0.80 (0.44)	(−1.69, 0.09)	0.08	−1.33 (0.57)	(−2.46, −0.20)	0.02
Gender (reference: sale)				−1.31 (0.37)	(−2.04, −0.58)	0.001
Serving size × gender				0.82 (0.55)	(−0.28, 1.92)	0.14
AUDIT				0.07 (0.02)	(0.02, 0.11)	0.003
TRI Restrict				−0.02 (0.02)	(−0.06, 0.03)	0.47
Random components						
Level 2 variance (pairs)	2.48 (0.53)			1.84 (0.41)		
Level 1 variance (participants)	0.63 (0.12)			0.57 (0.11)		

AUDIT = Alcohol Use Disorders Identification Test. AUDIT scores range between 0 and 40. TRI = Temptation and Restraint Inventory. TRI Restrict scores range between 3 and 21. CI = confidence interval; SE = standard error.

### Perceived normality of serving size

On average, participants considered the provided serving sizes and the amount of alcohol that they consumed to be relatively ‘normal’ (average score greater than 3 out of 5). There was a trend for participants to perceive the smaller servings as less normal [B = −0.35 (−0.77, 0.06), SE = 0.21, *t*_(57)_ = 1.70, *P* = 0.10], but participants in both conditions perceived their own alcohol consumption during the study as comparably normal [B = −0.11 (−0.53, 0.30), SE = 0.21, *t*_(57)_ = 0.54, *P* = 0.59]. See Table 4 for estimated means.

**Table 4:** Study 1: perceived normality of the amount of alcohol participants personally consumed during the study and the serving size provided in the standard and reduced serving size conditions; means are estimated from multi‐level regression model accounting for data clustering within participant pairs.

	Serving size condition				
	Reduced (n = 60)		Standard (n = 54)		
	Mean (SE)	(95% CI)	Mean (SE)	(95% CI)	d
Normality of amount consumed during study	3.80 (0.15)	(3.50, 4.10)	3.68 (0.14)	(3.40, 3.97)	0.11
Normality of serving size	3.70 (0.15)	(3.40, 4.01)	3.35 (0.14)	(3.06, 3.64)	0.32

Perceived normality was measured on a 5‐point Likert scale, with greater scores indicating greater perceived normality.

## Study 2

We conducted study 2 to investigate the effect of serving size over a longer drinking period in a real‐world drinking environment.

## Methods

### Design

Participants attended one of four quiz nights in a bar in the centre of Liverpool. We allocated nights randomly so that standard‐sized beverages would be served on 2 nights and reduced‐sized beverages on 2 nights. The unadjusted analysis in study 1 showed a medium effect size (*d* = 0.45, Supporting information, Table **S1**). Based on this, we needed a sample size of *n* = 128 to detect a medium effect (*d* = 0.50) in a two‐tailed *t*‐test (α = 0.05) with 80% power. To allow for dropouts and no‐shows, we recruited up to the bar's capacity (*n* = 50 per night, total *n* = 200). We used a cluster‐randomized design to ensure that participants were blind to the experimental manipulation. We measured how much alcohol participants consumed during the quiz (up to 3 hours) and participants later self‐reported any further alcohol consumption during the same evening. This permitted us to examine whether any reduction in alcohol consumption was subsequently compensated for.

### Participants

One hundred and sixty‐six participants attended one of the quiz nights. Participants were recruited in teams of two to five participants from the Liverpool area (e.g. on local social media pages, local radio, mailing lists from local organizations). Participants were eligible to take part if they were aged 18 years or older. The study received ethical approval from the University of Liverpool ethics committee. Testing took place in April and May 2017.

### Serving size manipulation

Quiz nights were assigned to a standard serving size condition or a reduced serving size condition in a counterbalanced order. Four types of beer/cider (average 4.85% ABV) and three types of wine (average 12% ABV) were available to purchase each night. On nights allocated to the standard serving size condition, beer and cider were served in non‐branded pint glasses (568 ml; 2.75 UK units/serving) and wine was served in 175‐ml servings in medium wine glasses (2.19 UK units/serving). On nights allocated to the reduced serving size condition, beer and cider were served in non‐branded ⅔ pint glasses (379 ml; 1.84 UK units/serving) and wine in 125‐ml servings in small wine glasses (1.50 UK units/serving). Participants could also order a variety of soft drinks, which were served in the same type of glass as beer and cider. The cost of each beverage was proportional to serving size and displayed near the bar.

### Observing alcohol consumption

Researchers recorded individual participants’ alcohol consumption covertly during the quiz. Additionally, one member of staff serving at the bar and one researcher recorded the total number of beverages that were sold on each night and the amount of wastage. All observers were aware of the study hypotheses. See Supporting information for a detailed description of observation methods.

### Procedure

The study and analysis protocol was registered at http://osf.io/2tmu6 prior to data collection. Testing took place in the function room of a local bar on Tuesday and Wednesday evenings between 19:30 and 22:30 hours. To obscure the real aims of the study, participants were informed that they would be taking part in a study investigating how personality characteristics affected group performance in a quiz. Upon arrival, participants gave verbal consent, provided their age and gender and completed a short bogus personality questionnaire. Participants were asked to purchase beverages only from the private bar in the function room and not from the bar's main area. 1The main bar served different beverage types than the private bar for the experiment, and beverages from the main bar were not included in the serving size manipulation. Unexpectedly, 18 participants (all in the reduced serving size condition) purchased beverages from the main bar in the pub during the pub quiz (which was not subject to the serving size manipulation). Beverages purchased from the main bar were not included in the observed alcohol consumption score used in the primary analyses (per pre‐registration protocol). We conducted two exploratory sensitivity analyses to investigate how this affected our main results. The results followed the same pattern as the primary analyses (see Supporting information, Tables S6–S9). Unadjusted analyses showed that participants consumed 23.2–31.9% less alcohol in the reduced serving size versus standard size condition. Adjusted analyses controlling for gender, AUDIT‐C scores and self‐reported alcohol consumption prior to the quiz were not significant. The quiz lasted approximately 1 hour and 40 minutes (see Supporting information). Participants could arrive up to 30 minutes prior to the quiz and were asked to leave 45 minutes after the quiz ended (a minimum of 1 hour and 40 minutes and a maximum of 3 hours to order and consume beverages).

The following day, participants completed an online questionnaire that included the AUDIT‐C 20 as a measure of typical alcohol consumption. Participants also reported the brand/type and serving size of any alcoholic beverages they consumed before, during and after the quiz. We used the brand and serving size information to calculate the number of UK units in each beverage. If participants were not able to remember the exact brand they consumed, we calculated the number of UK units based on the average ABV for each beverage type 21. To corroborate the cover story, these measures were embedded in questionnaires about participants’ contribution to their quiz team. Responses to the follow‐up questionnaire that were submitted more than 7 days after the quiz were excluded from analysis due to concerns about reduced recall accuracy 22. All participants were debriefed and informed about the real aims of the study 7 days after the final quiz night took place.

### Data analysis

#### Observed alcohol consumption during the quiz

We used multi‐level regression modelling to evaluate the amount of alcohol consumed across conditions, while controlling for data clustering within teams and quiz nights. In an adjusted analysis, we also controlled for gender (between‐subjects factor), AUDIT‐C scores and self‐reported alcohol consumption prior to the quiz (covariates). Because observed alcohol consumption was not distributed normally, we created 1000 bootstrap samples to estimate bias‐corrected and accelerated 95% confidence intervals (CIs) for the model parameters [bias‐corrected and accelerated (BCa) 95% CIs]. Analyses not accounting for clustering are reported in the Supporting information, Table S5.

#### Self‐reported alcohol consumption after the quiz

To examine whether participants would compensate for the reduced servings by consuming more alcohol after the study, we analysed the effect of serving size condition on self‐reported alcohol consumption after the quiz using a Bayesian *t*‐test for independent samples. We also analysed the effect of serving size condition on self‐reported alcohol consumption after the quiz, while controlling for gender, AUDIT‐C scores and self‐reported alcohol consumption prior to the quiz using a Bayesian analysis of covariance (ANCOVA). We used Bayesian analysis because we hypothesized that the serving size manipulation would not affect consumption significantly after the study 23. Bayesian analyses were conducted in JASP version 0.8.1.1 24. All other analyses were conducted in SPSS version 24 19.

## Results

### Participant characteristics

As per study protocol, two participants were excluded from all analyses because they guessed that their alcohol consumption was being observed during the study. The final sample consisted of 164 participants (see Table 5). One participant did not complete the questionnaire during the study. Sixteen participants did not complete the follow‐up questionnaire. These participants were excluded where applicable using listwise exclusion.

**Table 5:** Study 2: participant characteristics by serving size condition.

		Serving size condition	
	Total (N = 164)	Reduced (n = 87)	Standard (n = 77)
Gender^a^; *n* male/female	69/94	36/50	33/44
Number of individual teams	38	19	19
Team size; mean (SD)	4.37 (0.98)	4.63 (0.74)	4.11 (1.12)
Age^a^; mean (SD)	34.89 (12.45)	34.57 (11.58)	35.25 (13.42)
AUDIT‐C^b^; mean (SD)	4.43 (1.82)	4.26 (1.84)	4.64 (1.79)
Self‐reported consumption before study (UK units)^b^; Mean (SD)	1.75 (2.11)	2.04 (2.22)	1.36 (1.91)
Attrition; % lost to follow‐up	9.76%	3.45%	16.88%

AUDIT = Alcohol Use Disorders Identification Test; AUDIT‐C scores range between 0 and 12.

a One participant did not complete the demographics questionnaire. Statistics for these variables are based on total *N* = 163 (reduced *n* = 86, standard *n* = 77).

b AUDIT‐C and self‐reported consumption before the study were measured in the follow‐up questionnaire. Means and standard deviations (SDs) for these variables are based on total *N* = 148 (reduced *n* = 84, standard *n* = 64).

### Observed alcohol sales

The bar sold on average 28.07% less alcohol on nights with reduced servings (mean = 77.9 UK units, SD = 14.3) than on nights with standard servings (mean = 108.3 UK units, SD = 6.1; means weighted for number of attendees).

### Observed alcohol consumption

The results of the multi‐level modelling showed a significant reduction in alcohol consumption attributed to the reduced serving size condition [B = −1.14 (−1.68, −0.60), standard error (SE) = 0.28, *P* = 0.001]. However, this reduction [B = −0.73 (−1.78, 0.27), SE = 0.52, *P* = 0.14] became non‐significant when controlling for gender, AUDIT‐C scores and self‐reported alcohol consumption prior to the quiz (Table 6). Inspection of the estimated means shows that participants in the reduced serving size condition drank 32.4–39.6% less alcohol than participants in the standard serving size condition (Supporting information, Table S4, Fig. 2).

**Table 6:** Study 2: unadjusted and adjusted multi‐level regression model with serving size predicting observed alcohol consumption (UK units); participants are clustered in teams (level 2) and quiz nights (level 3).

	Unadjusted (n = 164)			Adjusted (n = 148)		
	B (SE)	(BCa 95% CI)	P	B (SE)	(BCa 95% CI)	P
Fixed components						
Intercept	2.88 (0.23)	(2.41, 3.36)	0.001	1.59 (0.53)	(0.43, 3.07)	0.004
Serving size condition (reference: standard)	−1.14 (0.28)	(−1.68, −0.60)	0.001	−0.73 (0.52)	(−1.78, 0.27)	0.14
Gender (reference: male)				−0.54 (0.57)	(−1.59, 0.42)	0.35
Serving size × gender				−0.31 (0.69)	(−1.68, 1.05)	0.66
AUDIT‐C				0.30 (0.08)	(0.14, 0.44)	0.001
Consumption before quiz				0.03 (0.10)	(−0.12, 0.17)	0.73
Random components						
Level 3 × 2 variance (quiz night × teams)	1.38 (0.37)			1.17 (0.37)		
Level 1 variance (participants)	2.84 (0.35)			2.23 (0.28)		

AUDIT = Alcohol Use Disorders Identification Test; AUDIT‐C scores range between 0 and 12. CI = confidence interval; SE = standard error; BCa = bias‐corrected and accelerated.

### Self‐reported alcohol consumption after the study

The Bayesian analysis was inconclusive in an unadjusted analysis [Bayes factor (BF)_10_ = 0.36]. However, after controlling for gender, AUDIT‐C scores and self‐reported consumption before the study, there was sufficient evidence that serving size condition did not affect self‐reported alcohol consumption after the study (BF_10_ = 0.29). See Table 7 for (un)adjusted means.

**Table 7:** Study 2: unadjusted and adjusted mean self‐reported alcohol consumption (UK units) after the quiz in the standard and reduced serving size condition.

	Unadjusted			Adjusted^a^		
Serving size condition	Mean (SD)	(95% CI)	BF_10_	Mean (SE)	(95% CI)	BF_10_
Standard (*n* = 64)	1.36 (2.58)	(0.71, 2.00)	0.36	1.36 (0.24)	(0.88, 1.84)	0.29
Reduced (*n* = 84)	0.92 (1.74)	(0.54, 1.30)		1.02 (0.21)	(0.60, 1.44)	

a Means adjusted for gender, AUDIT‐C scores and self‐reported alcohol consumption before the quiz. CI = confidence interval; SD = standard deviation; BF = Bayes factor.

## Policy model

We used the SAPM version 3.1 15 to estimate the potential effect of systematic reductions in the serving size of all beverages served in the on‐trade on alcohol‐related harm. As the effect size in study 2 was substantially larger than in study 1, we based the policy model on the more conservative effect size from study 1.

SAPM is a deterministic mathematical simulation model that models how alcohol policies such as pricing and taxation changes 25 affect alcohol consumption and the resulting changes in alcohol‐attributable mortality and morbidity. The model methodology is described extensively elsewhere 15, 26. SAPM uses alcohol consumption data from the 2014 Health Survey for England (HSE) to represent baseline consumption in the model. These data are combined with alcohol purchasing data from the 2010–14 Living Costs and Food Surveys to estimate the proportion of each HSE respondent's consumption, which falls into 10 categories: on‐ and off‐trade beer, cider, wine, spirits and Ready‐to‐Drinks (RTDs) (pre‐mixed beverages often referred to as ‘alcopops’)—see Brennan *et al*. [27] for full details of this apportionment process. Based on the results of study 1 we estimated that a 25% reduction in serving size could lead to an approximate reduction in alcohol consumption of 20.7%. We modelled the effect of this reduction in on‐trade alcohol consumption, as well as a more conservative estimate (a 10.3% decrease—half the effect size in study 1), to account for the possibility that the population effect size is substantially smaller. See Supporting information for full details. For each scenario we modelled the long‐term (20‐year 27) impact on alcohol‐attributable deaths and hospital admissions from 43 different alcohol‐related health conditions and compared these to a counterfactual scenario where alcohol consumption remained unchanged. The modelled scenarios resulted in an estimated 5.6–13.2% reduction in deaths per year and an estimated 4.4–10.5% reduction in hospital admissions per year compared to the baseline scenario (see Table 8).

**Table 8:** Policy model: annual effects of a 25% reduction in the serving size of alcohol sold in the on‐trade on alcohol‐related deaths and hospital admissions, compared to a ‘no policy’ baseline model, 20 years after policy implementation.

Policy scenario	Deaths per year		Hospital admissions per year	
	Absolute	Relative	Absolute	Relative
Baseline	12 284		833 722	
(1) 20.7% reduction in all on‐trade alcohol consumption	−1616	−13.16%	−87 853	−10.54%
(2) 20.7% reduction on on‐trade beer, cider and wine consumption only	−1360	−11.07%	−73 244	−8.79%
(3) 10.3% reduction in all on‐trade alcohol consumption	−819	−6.67%	−44 021	−5.28%
(4) 10.3% reduction in on‐trade beer, cider and wine consumption only	−687	−5.59%	−36 650	−4.40%

## Discussion

We investigated the effect of alcoholic beverage serving size on alcohol consumption. In study 1, we demonstrated that reduced serving sizes led to a 20.7–22.3% decrease in alcohol consumption in the laboratory during a 1‐hour drinking period. In study 2, we showed that reduced serving sizes led to a 32.4–39.6% decrease in alcohol consumption in a real‐world drinking environment during a longer drinking period (up to 3 hours). Additional sensitivity analyses indicated a reduction of 17.4–31.9% attributed to the reduced serving size. People did not compensate for the serving size reductions by consuming more alcohol after the study. These findings support our hypothesis that serving size has a causal effect on alcohol consumption.

The exact magnitude of the reduction in alcohol consumption was dependent upon the analysis used. The analysis adjusting for clustering of alcohol consumption within participant pairs/teams showed a 20.7 and 39.6% reduction in alcohol consumption in studies 1 and 2, respectively. Because clustering occurred in both studies, we believe this to be the best approximation of the effect of serving size on alcohol consumption in the present studies.

While the reduction in serving size led to a reduction in alcohol consumption in both studies, the reduction in alcohol consumption was somewhat larger in study 2 (where standard serving sizes of 2.8 units for beer/cider and 2.2 units for wine were reduced by 28.7–33.3%) than in study 1 (where standard serving sizes of 2.1 units were reduced by 25%). One explanation is that greater serving size reductions will prompt greater reductions in alcohol consumption. However, given the differences between the two study designs, other factors may partially explain this difference. A further difference between the two studies is that in study 1 participants were required to consume at least some alcohol and had access only to alcoholic beverages, whereas participants in study 2 were able to consume non‐alcoholic beverages and were not required to drink any alcohol at all.

These studies are the first, to our knowledge, to demonstrate that reducing the serving size of alcoholic beverages prompts reductions in alcohol consumption. This is consistent with research demonstrating that food portion size has a causal effect on energy intake 28 and consumers do not fully compensate for the effect that portion size has on total energy intake 10, 29. In the present studies we examined alcohol consumption during relatively short periods. However, in study 2 we found that reduced serving sizes led to decreased alcohol consumption during 3 hours; a length of time that is comparable to most drinking occasions in the UK population 30. In study 2 we also found no evidence that participants consumed more alcohol during the remainder of the night if they had been provided earlier with reduced serving size alcoholic beverages. It would now be informative for future research to investigate the long‐term effect of reducing the standard serving sizes of alcoholic beverages on alcohol consumption.

As the aim of the present research was to examine the causal influence of serving size on alcohol consumption, we did not make participants explicitly aware of the serving size reductions made. Instead, we used cover stories in both experiments, limiting the likelihood that our findings can be explained by demand characteristics 31. We cannot completely rule out demand characteristics in either study, because participants in the reduced serving size conditions may have been conscious of the fact that they were receiving a smaller than usual serving of alcohol. If serving size reductions to on‐trade alcoholic beverages were to be implemented as a policy, this would require transparency. It is possible that unfavourable opinions towards systematic alcohol serving size reductions would lead to psychological reactance to the policy 32, limiting its effectiveness. It would therefore be informative to examine public acceptability of serving size reductions to alcoholic beverages and whether awareness of serving size reductions affects their influence on alcohol consumption.

Our methodology had some limitations. First, observers in study 2 were aware of the study aims. This could have influenced the way they coded alcohol consumption. However, in line with recommendations 33, the observers were well trained and each participant was observed by two independent observers. Secondly, glass size varied between serving size conditions to ensure that glasses appeared similarly full. People may consume more alcohol from larger glasses 14, 34. Therefore, future work may benefit from controlling for glass size when examining the effect of serving size. Thirdly, participants in study 1 were primarily university students and despite recruiting from the local community for study 2, the sample may not be representative of the UK population. Future research should investigate the effect of serving size reduction in more diverse populations and examine whether the effect is moderated by demographic characteristics.

The typical serving size of beer in the United Kingdom (568 ml) is larger than serving sizes used in many other countries 35, and the size of on‐trade wine servings in the United Kingdom has increased during recent decades 36. It is therefore feasible that existing serving size legislation 37 could be adapted to introduce a cap on available serving sizes and accommodate the sales of smaller servings. We used the SAPM to estimate the potential public health benefit of reducing the default serving sizes of alcoholic beverages in the on‐trade. Our most conservative estimates suggest that serving size reductions might reduce alcohol‐related deaths and hospital admissions to a similar extent as a £0.50 minimum unit price 15. However, it is important to acknowledge that these estimates are subject to limitations of our studies outlined above. Additionally, while the aggregate effects of serving size reductions and minimum unit pricing may be similar, the cheap alcohol that would be affected by minimum unit pricing is consumed by different demographics than the on‐trade alcohol that would be affected by serving size reductions. Therefore, the distribution of effects would probably be very different for both policies. Nevertheless, our findings highlight alcoholic beverage serving size as a potential target for public health interventions.

To conclude, this research is the first to demonstrate that the serving size of alcoholic beverages affects alcohol consumption. Reducing the standard serving sizes of alcoholic beverages may be an effective way to reduce alcohol consumption at the population level.

## Declaration of interests

None.

## Supporting information


**Table S1** Unadjusted and adjusted mean observed alcohol consumption (UK units) in the standard and reduced serving size condition. Analyses are not adjusted for clustering.
**Table S2** Perceived normality of the amount of alcohol participants personally consumed during the study and the serving size provided in the standard and reduced serving size conditions. Analyses are not adjusted for clustering.
**Table S3** Unadjusted and adjusted mean observed alcohol consumption (UK units) in the standard and reduced serving size condition. Means are estimated from multi‐level regression model accounting for data clustering within participant pairs (Table 3).
**Table S4** Unadjusted and adjusted mean observed alcohol consumption (UK units) in the standard and reduced serving size condition. Means are estimated from multi‐level regression model accounting for data clustering within teams and quiz nights (Table 6).
**Table S5** Unadjusted and adjusted mean observed alcohol consumption (UK units) in the standard and reduced serving size condition.
**Table S6** Exploratory analysis. Unadjusted and adjusted multi‐level regression model with serving size predicting observed alcohol consumption (UK units). Participants are clustered within teams (level 2) and quiz nights (level 3), excluding any participants who consumed drinks from the main bar.
**Table S7** Exploratory analysis. Unadjusted and adjusted mean observed alcohol consumption (UK units) in the standard and reduced serving size condition, excluding any participants who consumed drinks from the main bar. Means are estimated from multi‐level regression model accounting for data clustering within teams and quiz nights (Table S1).
**Table S8** Exploratory analysis. Unadjusted and adjusted multi‐level regression model with serving size predicting observed alcohol consumption (UK units). Participants are clustered in teams (level 2) and quiz nights (level 3). The content of drinks that were purchased downstairs was estimated from observer records and self‐reported consumption.
**Table S9** Exploratory analysis. Unadjusted and adjusted mean observed alcohol consumption (UK units) in the standard and reduced serving size condition. The content of drinks that were purchased downstairs was estimated from observer records and self‐reported consumption. Means are estimated from multi‐level regression model accounting for data clustering within teams and quiz nights (Table S3).
