# Supplementary material for: Reducing the standard serving size of alcoholic beverages prompts reductions in alcohol consumption
Source: Addiction. 2018 May 14;113(9):1598–608. doi: 10.1111/add.14228 (PMC6099514; doi:10.1111/add.14228)
Supplement: Supplementary file 1 — Table S1 Unadjusted and adjusted mean observed alcohol consumption (UK units) in the standard and reduced serving size condition. Analyses are not adjusted for clustering. Table S2 Perceived normality of the amount of alcohol participants personally consumed during the study and the serving size provided in the standard and reduced serving size conditions. Analyses are not adjusted for clustering. Table S3 Unadjusted and adjusted mean observed alcohol consumption (UK units) in the standard and reduced serving size condition. Means are estimated from multi‐level regression model accounting for data clustering within participant pairs (Table 3). Table S4 Unadjusted and adjusted mean observed alcohol consumption (UK units) in the standard and reduced serving size condition. Means are estimated from multi‐level regression model accounting for data clustering within teams and quiz nights (Table 6). Table S5 Unadjusted and adjusted mean observed alcohol consumption (UK units) in the standard and reduced serving size condition. Table S6 Exploratory analysis. Unadjusted and adjusted multi‐level regression model with serving size predicting observed alcohol consumption (UK units). Participants are clustered within teams (level 2) and quiz nights (level 3), excluding any participants who consumed drinks from the main bar. Table S7 Exploratory analysis. Unadjusted and adjusted mean observed alcohol consumption (UK units) in the standard and reduced serving size condition, excluding any participants who consumed drinks from the main bar. Means are estimated from multi‐level regression model accounting for data clustering within teams and quiz nights (Table S1). Table S8 Exploratory analysis. Unadjusted and adjusted multi‐level regression model with serving size predicting observed alcohol consumption (UK units). Participants are clustered in teams (level 2) and quiz nights (level 3). The content of drinks that were purchased downstairs was estimated from observer records and se [file ADD-113-1598-s001.docx]

**Results study 1**

**Alcohol consumption**

We used an independent samples t-test to evaluate the amount of alcohol consumed across conditions in an unadjusted analysis, and in an adjusted analysis we compared conditions using an ANCOVA to control for gender (between-subjects factor), AUDIT-c scores, and TRI Restrict scores (covariates).

Participants in the reduced serving size condition drank significantly less alcohol than participants in the standard serving size condition in both the unadjusted and adjusted main analyses (See Table S1).

| **Table S1.** Study 1. Unadjusted and adjusted mean observed alcohol consumption (UK units) in the standard and reduced serving size condition. Analyses are not adjusted for clustering. | | | | | | | | |
| --- | --- | --- | --- | --- | --- | --- | --- | --- |
|  | Unadjusted | | | | Adjusted^a^ | | | |
| Serving size condition | Mean (SE) | t(112) | p | d | Mean (SE) | F(1,108) | p | η_p_^2^ |
| Standard (n = 60) | 3.87 (0.26) | 2.40 | 0.02 | 0.45 | 4.19 (0.23) | 6.07 | 0.001 | 0.05 |
| Reduced (n = 54) | 3.07 (0.22) |  |  |  | 3.32 (0.27) |  |  |  |
| Difference | -0.80 (0.33) |  |  |  | -0.87 (0.35) |  |  |  |
| Relative difference^b^ | -20.67% |  |  |  | -20.76% |  |  |  |
| Note: ^a^ Means adjusted for gender, AUDIT scores and TRI Restrict scores. ^b^ Relative difference in alcohol consumption in the reduced compared to standard serving size condition. | | | | | | | | |

**Perceived normality of serving size**

We conducted independent samples t-tests to compare the two serving size conditions on perceived normality of the serving size and the amount of alcohol that participants consumed during the study.

On average, participants considered the provided serving sizes and the amount of alcohol that they consumed to be relatively ‘normal’ (average score greater than 3 out of 5) and the two conditions did not significantly differ on these measures (Table S2). However, servings were considered to be marginally less normal if they were reduced compared to standard size (p = .06).

| **Table S2.** Study 1. Perceived normality of the amount of alcohol participants personally consumed during the study and the serving size provided in the standard and reduced serving size conditions. Analyses are not adjusted for clustering. | | | | | |
| --- | --- | --- | --- | --- | --- |
|  | Serving size condition | |  |  |  |
|  | Reduced (*n* = 60) | Standard (*n* = 54) |  |  |  |
|  | Mean (SD) | Mean (SD) | t(112) | p | d |
| Normality of amount consumed during study | 3.68 (.93) | 3.80 (.88) | -.67 | .51 | .13 |
| Normality of serving size | 3.35 (1.05) | 3.70 (.90) | -1.91 | .06 | .36 |
| *Note:* Perceived normality was measured on a 5-point Likert scale, with greater scores indicating greater perceived normality. | | | | | |

**Methods study 2**

**Observing alcohol consumption**

*Individual alcohol consumption:* Six researchers posed as pub quiz participants and covertly observed participants’ alcohol consumption during the quiz. Each participant was observed by two researchers. Every time a participant ordered a new drink, each researcher independently recorded the type of drink (cider/beer, wine, soda). If participants did not consume their entire drink, the researchers estimated how much of it was left rounded to the nearest quarter (i.e., estimating whether one quarter, half, three quarters or all of the drink was left in the glass). We calculated how many alcohol units each participant consumed using 4.85% ABV for each observed drink of cider/beer (2.75 UK units in standard and 1.84 UK units in reduced condition) and 12% ABV for each observed drink of wine (2.1 UK units in standard and 1.5 UK units in reduced condition), subtracting any observed waste. For each participant, we took the average of the observed alcohol consumption from the two observing researchers. Interrater agreement was high (ICC = 0.96).

*Total alcohol purchased:* One member of staff serving at the bar and one researcher recorded how many glasses of each available drink were sold on each night, and measured how much alcohol was left in glasses that were returned to the bar (wastage). We took the average of the two observers as the total amount of alcohol units sold on each night. Interrater agreement was high (ICC = 0.99).

**Pub quiz procedures**

The quiz had three distinct question rounds (round 1: Arts and Literature; round 2: Sports; round 3: Science and Nature), starting at 30 minute intervals and two paper-pencil question sheets (“Guess the film” and “Song lyrics”) for teams to complete during the breaks between the question rounds. After the last break, the answers to the questions were announced and participants marked another team’s answer sheets. The winning team was announced approximately 1 hour and 40 minutes after the start of the quiz and received a £50 prize. The doors opened 30 minutes prior to the quiz and participants were allowed to stay until 45 minutes after the announcement of the scores. All participants therefore had a minimum of 1 hour and 40 minutes and a maximum of 3 hours to order and consume drinks.

**Supplementary Tables study 2**

| **Table S3.** Study 1. Unadjusted and adjusted mean observed alcohol consumption (UK units) in the standard and reduced serving size condition. Means are estimated from multilevel regression model accounting for data clustering within participant pairs (Table 3). | | | | | | |
| --- | --- | --- | --- | --- | --- | --- |
|  | Unadjusted | | | Adjusted^a^ | | |
| Serving size condition | Mean (SE) | [95% CI] | d | Mean (SE) | [95% CI] | d |
| Standard (*n* = 60) | 3.87 (0.32) | [3.23, 4.52] | 0.34 | 4.12 (0.29) | [3.54, 4.69] | 0.42 |
| Reduced (*n* = 54) | 3.07 (0.31) | [2.46, 3.68] |  | 3.20 (0.30) | [2.61, 3.79] |  |
| Difference | -0.80 (0.44) | [-1.69, .09] |  | -0.92 (0.41) | [-1.74, -0.09] |  |
| Relative difference^b^ | -20.7% |  |  | -22.3% |  |  |
| Note: ^a^ Means adjusted for gender, AUDIT scores and TRI Restrict scores. ^b^ Relative difference in alcohol consumption in the reduced compared to standard serving size condition. | | | | | | |

| **Table S4.** Study 2. Unadjusted and adjusted mean observed alcohol consumption (UK units) in the standard and reduced serving size condition. Means are estimated from multilevel regression model accounting for data clustering within teams and quiz nights (Table 6). | | | | | | | | |
| --- | --- | --- | --- | --- | --- | --- | --- | --- |
|  | Unadjusted | | | | Adjusted^a^ | | | |
| Serving size condition | Mean (SE) | [95% CI] | *n* | d | Mean (SE) | [95% CI] | *n* | d |
| Standard | 2.88 (0.33) | [2.21, 3.56] | 77 | 0.38 | 2.72 (0.32) | [2.07, 3.37] | 64 | 0.33 |
| Reduced | 1.74 (0.33) | [1.08, 2.40] | 87 |  | 1.84 (0.30) | [1.23, 2.45] | 84 |  |
| Difference | -1.14 (0.47) | [-2.08, -0.20] |  |  | -0.88 (0.44) | [-1.78, 0.02] |  |  |
| Relative difference^b^ | -39.6% |  |  |  | -32.4% |  |  |  |
| Note: ^a^ Means adjusted for gender, AUDIT-c scores and self-reported alcohol consumption before the quiz. ^b^ Relative difference in alcohol consumption in the reduced compared to standard serving size condition. | | | | | | | | |

**Unadjusted analyses study 2**

First, we analysed the effect of serving size condition on observed alcohol consumption during the quiz using Welch’s t-test for independent samples. Then, we used an ANCOVA to analyse the effect of serving size condition on observed alcohol consumption, whilst controlling for gender (between-subjects factor), AUDIT-c scores, and self-reported alcohol consumption prior to the pub quiz (covariates). Because observed alcohol consumption was not normally distributed, we created 1000 bootstrap samples to estimate bias-corrected and accelerated 95% confidence intervals (BCa 95% CIs).

Participants who were served reduced serving sizes drank significantly less alcohol during the study than participants who were served standard serving sizes (see Table S5).

| **Table S5.** Study 2. Unadjusted and adjusted mean observed alcohol consumption (UK units) in the standard and reduced serving size condition. | | | | | | | | | | |
| --- | --- | --- | --- | --- | --- | --- | --- | --- | --- | --- |
|  | Unadjusted | | | | | Adjusted^a^ | | | | |
| Serving size condition | M (SE) | BCa 95% CI | n | p | d | M (SE) | BCa 95% CI | n | p | η_p_^2^ |
| Standard | 2.93 (0.25) | [2.47, 3.42] | 77 | 0.001 | .61 | 2.81 (0.24) | [2.30, 3.29] | 64 | .009 | .06 |
| Reduced | 1.70 (0.20) | [1.32, 2.13] | 87 |  |  | 1.82 (0.21) | [1.36, 2.24] | 84 |  |  |
| Difference | -1.23 (0.31) | [-1.89, -0.57] |  |  |  | -0.99 (0.34) | [-1.66, -0.41] |  |  |  |
| Relative difference^b^ | -41.98% |  |  |  |  | -35.23% |  |  |  |  |
| Note: ^a^ Means adjusted for gender, AUDIT-c scores and self-reported alcohol consumption before the quiz. ^b^ Relative difference in alcohol consumption in the reduced compared to standard serving size condition. | | | | | | | | | | |

**Exploratory analyses study 2**

*Exploratory analysis*

First, we excluded any participants who purchased drinks from the main bar. The analysis unadjusted for covariates showed a significant reduction in alcohol consumption attributed to the reduced serving size condition (B = -0.92 [-1.55, -0.30], SE = 0.32, p = 0.002). However, this reduction became non-significant when controlling for gender, AUDIT-c scores, and self-reported alcohol consumption prior to the quiz (B = -0.38 [-1.54, 0.47], SE = 0.57, p = 0.43) (Table S6). Inspection of the estimated means shows that participants in the reduced serving size condition drank 22.3%-31.9% less alcohol than participants in the standard serving size condition (Table S7).

Then, we included the drinks ordered from the main bar in the observed alcohol consumption score, based on observer records and participant self-reported alcohol consumption. The analysis unadjusted for covariates showed a significant reduction in alcohol consumption attributed to the reduced serving size condition (B = -0.67 [-1.28, -0.08], SE = 0.30, p = 0.007). However, this reduction became non-significant when controlling for gender, AUDIT-c scores, and self-reported alcohol consumption prior to the quiz (B = -0.18 [-1.20, 0.94], SE = 0.51, p = 0.68) (Table S8). Inspection of the estimated means shows that participants in the reduced serving size condition drank 17.4%-23.2% less alcohol than participants in the standard serving size condition (Table S9).

| **Table S6.** Study 2. Exploratory analysis. Unadjusted and adjusted multilevel regression model with serving size predicting observed alcohol consumption (UK units). Participants are clustered within teams (level 2) and quiz nights (level 3), excluding any participants who consumed drinks from the main bar. | | | | | | |
| --- | --- | --- | --- | --- | --- | --- |
|  | Unadjusted (*n* = 146) | | | Adjusted (*n* = 132) | | |
|  | B (SE) | [BCa 95% CI] | p | B (SE) | [BCa 95% CI] | p |
| Fixed components | | | | | | |
| Intercept | 2.88 (0.23) | [2.46, 3.33] | 0.001 | 1.45 (0.60) | [0.28, 3.13] | 0.01 |
| Serving size condition (reference: Standard) | -0.92 (0.32) | [-1.54, -0.30] | 0.001 | -0.38 (0.57) | [-1.54, 0.57] | 0.43 |
| Gender (reference: Male) |  |  |  | -0.52 (0.60) | [-1.66, 0.52] | 0.38 |
| Serving size x Gender |  |  |  | -0.46 (0.75) | [-1.99, 1.43] | 0.55 |
| AUDIT-c |  |  |  | 0.30 (0.09) | [0.14, 0.43] | .003 |
| Consumption before quiz |  |  |  | 0.15 (0.09) | [-0.02, 0.29] | 0.12 |
| Random components | | | | | | |
| Level 3*2 variance (quiz night * teams) | 1.56 (0.43) |  |  | 1.12 (0.44) |  |  |
| Level 1 variance (participants) | 2.92 (0.37) |  |  | 2.24 (0.31) |  |  |
| *Note:* AUDIT = Alcohol Use Disorders Identification Test. AUDIT-c scores range between 0 and 12. | | | | | | |

| **Table S7.** Study 2. Exploratory analysis. Unadjusted and adjusted mean observed alcohol consumption (UK units) in the standard and reduced serving size condition, excluding any participants who consumed drinks from the main bar. Means are estimated from multilevel regression model accounting for data clustering within teams and quiz nights (Table S1). | | | | | | | | |
| --- | --- | --- | --- | --- | --- | --- | --- | --- |
|  | Unadjusted | | | | Adjusted^a^ | | | |
| Serving size condition | Mean (SE) | [95% CI] | *n* | d | Mean (SE) | [95% CI] | *n* | d |
| Standard | 2.88 (0.35) | [2.17, 3.59] | 77 | 0.30 | 2.73 (0.32) | [2.08, 3.37] | 64 | 0.24 |
| Reduced | 1.96 (0.37) | [1.22, 2.70] | 69 |  | 2.12 (0.32) | [1.47, 2.77] | 68 |  |
| Difference | -0.92 (0.51) | [-1.95, 0.10] |  |  | -0.61 (0.45) | [-1.53, 0.31] |  |  |
| Relative difference^b^ | -31.9% |  |  |  | -22.3% |  |  |  |
| *Note:* ^a^ Means adjusted for gender, AUDIT-c scores and self-reported alcohol consumption before the quiz. ^b^ Relative difference in alcohol consumption in the reduced compared to standard serving size condition. | | | | | | | | |

| **Table S8.** Study 2. Exploratory analysis. Unadjusted and adjusted multilevel regression model with serving size predicting observed alcohol consumption (UK units). Participants are clustered in teams (level 2) and quiz nights (level 3). The content of drinks that were purchased downstairs was estimated from observer records and self-reported consumption. | | | | | | |
| --- | --- | --- | --- | --- | --- | --- |
|  | Unadjusted (*N* = 164) | | | Adjusted (*n* = 148) | | |
|  | B (SE) | [BCa 95% CI] | p | B (SE) | [BCa 95% CI] | p |
| Fixed components | | | | | | |
| Intercept | 2.89 (0.23) | [2.45, 3.33] | 0.001 | 1.24 (0.53) | [0.21, 2.40] | 0.02 |
| Serving size condition (reference: Standard) | -0.67 (0.30) | [-1.28, -0.08] | 0.007 | -0.18 (0.51) | [-1.20, 0.94] | 0.68 |
| Gender (reference: Male) | - |  |  | -0.49 (0.52) | [-1.56, 0.62] | 0.36 |
| Serving size x Gender | - |  |  | -0.61 (0.67) | [-1.96, 0.62] | 0.37 |
| AUDIT-c | - |  |  | 0.34 (0.08) | [0.16, 0.50] | 0.001 |
| Consumption before quiz | - |  |  | 0.14 (0.09) | [-0.009, 0.26] | 0.11 |
| Random components | | | | | | |
| Level 3*2 variance (quiz night * teams) | 1.31 (0.39) |  |  | 0.97 (0.35) |  |  |
| Level 1 variance (participants) | 3.15 (0.36) |  |  | 2.27 (0.29) |  |  |
| *Note:* AUDIT = Alcohol Use Disorders Identification Test. AUDIT-c scores range between 0 and 12. | | | | | | |

| **Table S9.** Study 2. Exploratory analysis. Unadjusted and adjusted mean observed alcohol consumption (UK units) in the standard and reduced serving size condition. The content of drinks that were purchased downstairs was estimated from observer records and self-reported consumption. Means are estimated from multilevel regression model accounting for data clustering within teams and quiz nights (Table S3). | | | | | | | | |
| --- | --- | --- | --- | --- | --- | --- | --- | --- |
|  | Unadjusted | | | | Adjusted^a^ | | | |
| Condition | M (SE) | [95% CI] | *n* | d | M (SE) | [95% CI] | *n* | d |
| Standard | 2.89 (0.34) | [2.21, 3.56] | 77 | 0.22 | 2.76 (0.31) | [2.14, 3.38] | 64 | 0.19 |
| Reduced | 2.22 (0.33) | [1.56, 2.88] | 87 |  | 2.28 (0.28) | [1.70, 2.85] | 84 |  |
| Difference | -0.67 (0.47) | [-1.62, 0.28] |  |  | -0.48 (0.42) |  |  |  |
| Relative difference^b^ | -23.2% |  |  |  | -17.4% |  |  |  |
| *Note:* ^a^ Means adjusted for gender, AUDIT-c scores and self-reported alcohol consumption before the quiz. ^b^ Relative difference in alcohol consumption in the reduced compared to standard serving size condition. | | | | | | | | |

**Policy model**

The effect of reduced serving sizes was modelled under four alternative scenarios based on the findings of study 1:

1. A 20.7% reduction in all on-trade alcohol consumption (base case)
2. A 20.7% reduction on on-trade beer, cider and wine consumption only
3. A 10.3% reduction in all on-trade alcohol consumption
4. A 10.3% reduction in on-trade beer, cider and wine consumption only

Scenarios 2-4 represent more conservative assumptions where consumption of on-trade spirits and RTDs is unchanged, as these were excluded from studies 1 and 2, and a 50% reduction in the observed effect size. Under each scenario we assumed no further behavioural response (i.e. we assumed off-trade alcohol consumption remained unchanged).
